# Detection of two dissimilar chronic wasting disease isolates in two captive Rocky Mountain elk (*Cervus canadensis*) herds: Two distinctive chronic wasting disease isolates identified in captive elk

**DOI:** 10.1080/19336896.2021.1982333

**Published:** 2021-12-16

**Authors:** Tracy A. Nichols, Eric M. Nicholson, Yihui Liu, Wanyun Tao, Terry R. Spraker, Michael Lavelle, Justin Fischer, Qingzhong Kong, Kurt C. VerCauteren

**Affiliations:** a Veterinary Services Cervid Health Program, United States Department of Agriculture, Animal and Plant Health Inspection Service, Fort Collins, Colorado, USA; b Us Department of Agriculture, Agricultural Research Service, Ames, Iowa, USA; c Departments of Pathology, Neurology, National Center for Regenerative Medicine, and National Prion Disease Pathology Surveillance Center, Case Western Reserve University, Cleveland, Ohio, USA; d Prion Research Center and the Department of Microbiology, Immunology and Pathology, College of Veterinary Medicine and Biomedical Sciences, Colorado State University Prion Research Center, Fort Collins, Colorado, USA; e Wildlife Services National Wildlife Research Center, United States Department of Agriculture, Animal and Plant Health Inspection Service, Fort Collins, Colorado, USA

**Keywords:** Cervus elaphus, cervus canadensis, chronic wasting disease, conformational stability, CWD, disease prevalence, elk, isolates, prion, strains

## Abstract

Chronic wasting disease (CWD) continues to spread in both wild and captive cervid herds in North America and has now been identified in wild reindeer and moose in Norway, Finland and Sweden. There is limited knowledge about the variety and characteristics of isolates or strains of CWD that exist in the landscape and their implications on wild and captive cervid herds. In this study, we evaluated brain samples from two captive elk herds that had differing prevalence, history and timelines of CWD incidence. Site 1 had a 16-year history of CWD with a consistently low prevalence between 5% and 10%. Twelve of fourteen naïve animals placed on the site remained CWD negative after 5 years of residence. Site 2 herd had a nearly 40-year known history of CWD with long-term environmental accrual of prion leading to nearly 100% of naïve animals developing clinical CWD within two to 12 years. Obex samples of several elk from each site were compared for CWD prion strain deposition, genotype in prion protein gene codon 132, and conformational stability of CWD prions. CWD prions in the obex from site 2 had a lower conformational stability than those from site 1, which was independent of prnp genotype at codon 132. These findings suggest the existence of different CWD isolates between the two sites and suggest potential differential disease attack rates for different CWD strains.

## Introduction

Transmissible spongiform encephalopathies (TSEs) are infectious neurodegenerative diseases, also referred to as prion diseases that affect a number of mammals, such as humans, sheep, goats, cattle, cervids, minks, and felines. Chronic wasting disease (CWD) is a prion disease of animals in the family Cervidae. Natural transmission of CWD has been detected thus far in deer, elk, reindeer, and moose, and the disease continues to expand in wild and captive populations throughout the United States and Canada [1,2]. CWD has also been detected in wild moose and reindeer in Norway, Finland and Sweden [3]. Although CWD has been well documented and studied since the 1960s, there is still much unknown about it [4]. One such aspect is the prevalence and identification of distinctive isolates or strains, of the disease within and among species.

Unlike strains in bacterial or viral diseases, the presence of strains in prion diseases is not determined by genetic variability or mutations within the infectious organism itself because the infectious agent is an aberrantly folded endogenous protein [5,6]. The term ‘strain’ is found in the prion disease literature to describe variations in clinical signs, incubation period, distribution of the misfolded prion (PrP^RES^) in the body, and the stability of the PrP^RES^ protein within the same species and TSE type [7–13]. Strains have been documented in other prion diseases (e.g., scrapie, transmissible mink encephalopathy, bovine spongiform encephalopathy, Creutzfeldt-Jakob disease) and significant evidence indicates they are present in CWD as well, but it remains unclear as to how many exist and how they present in cervids [11,14–19]. Laboratory studies utilizing transgenic mice suggest that multiple CWD strains likely exist [10,20,21].

Here, we report that CWD appears to behave differently in two spatially disjunct, naturally infected captive elk populations, which precipitated comparative biochemical analysis of the CWD prions in animals from the two sites. Our results from these analyses confirm that the CWD prions at these two sites are different.

## Methods and materials

### Study animals

Elk in this study were from two captive, CWD-positive herds in the western United States where disease exposure occurred through natural routes. The tissues examined from these herds were comprised of both archived fixed and archived frozen brain samples from individuals that were found to be positive for CWD by immunohistochemical analysis of the obex and/or medial retropharyngeal lymph nodes. Specifics on animals and study sites are described below.

#### Site 1

Site 1 housed approximately 35 captive elks at any 1 time on 4.7 ha over a sixteen-year period and had a consistent annual CWD prevalence rate of 5–10% (2–3 positive animals), as established by rectal biopsy and post-mortem testing. This location was devoid of vegetation and was composed of rocky terrain and exposed soil.

##### CWD isolate study animals

Brains from two CWD-infected terminal-stage adults from this site were subjected to biochemical analysis. Animal 1-A was a seven-year-old adult male, and animal 1-B was a five-year-old adult female (Table 1).

**Table 1.** Study animal location, ID, sex, and PrP genotypes in codon 132 (M. methionine; L. leucine) for CWD isolate evaluation

Animal Site and ID	Sex	Age	Genotype at codon 132
1-A	M	7 yr	ML
1-B	F	5 yr	MM
2-A	F	Adult	MM
2-B	F	Adult	MM
2-C	F	Adult	ML
2-D	F	Adult	ML

##### Introduction of naïve animals

The animals were housed and euthanized in accordance with the USDA National Wildlife Research Center IACUC committee requirements. Thirteen one-year-old CWD-naïve male and 1 one-year-old CWD-naïve female elk were introduced to Site 1 to evaluate direct and indirect CWD transmissions (Table 2). Elks were purchased from a captive, CWD-negative herd in Minnesota in an area not reported to have CWD in the wild. Upon arrival, all elk had blood drawn for genotyping at PrP codon 132 by the USDA Agricultural Research Service in Pullman, WA, and were tested by rectal biopsy immunohistochemistry (IHC) for CWD. Six males were placed in a 0.1-ha pen where CWD-positive animals had been previously housed to investigate the potential for indirect transmission of CWD. The pen was double-fenced to prevent nose-to-nose contact with the rest of the population. The remaining seven males were integrated into the established main population to investigate direct transmission; the female was co-housed with an 11-year-old female who was a long-standing member of the herd. Animals were ante mortem tested via rectal biopsy as previously described [22], once a year throughout the five-year study period. Any animals found to have positive PrP^RES^ IHC staining in the rectal biopsy sample were euthanized within a week of detection.

**Table 2.** Site 1 direct and indirect contact animals. group, ID, sex, incubation period (if applicable), CWD status at death, and PrP genotypes at codon 132 (M, methionine; L, leucine)

Site 1 Indirect Contact Pen Animal ID	Sex	Incubation period	CWD Status at death	Genotype at codon 132
S11	M	>5 years	Non-detect	ML
44 T	M	>5 years	Non-detect	MM
51 T	M	>5 years	Non-detect	MM
806 T	M	>5 years	Non-detect	MM
808 T	M	>5 years	Non-detect	MM
814 T	M	>5 years	Non-detect	MM
**Site 1 Direct Contact Pens Animal ID**	**Sex**	**Age**	**CWD Status at death**	**Genotype at codon 132**
S12	M	>5 years	Non-detect	LL
S13	F	1 year	Positive	MM
58 T	M	>5 years	Non-detect	MM
800 T	M	>5 years	Non-detect	MM
801 T	M	3 years	Positive	MM
802 T	M	>5 years	Non-detect	MM
803 T	M	>5 years	Non-detect	MM
840 T	M	>5 years	Non-detect	MM

#### Site 2

Site 2 maintained between 10 and 100 elks in eight separate 0.2-ha pens during a 60-year time period. Other cervids including mule deer, white-tailed deer, and moose were also held at the facility. Multiple natural exposure CWD studies were conducted at the facility, and animals were often allowed to reach end-stage disease for various research projects [4,23–26]. Elks were typically rotated between pens and alleyways to allow for periodic grazing but were often held in pens devoid of vegetation with exposed soil. CWD was first documented at Site 2 in 1979; however, earlier cases may have been missed prior to the discovery and characterization of the disease. Annual prevalence rates were not established for this site in the same manner as Site 1. However, mortality testing revealed a significantly higher incidence of CWD than at Site 1. Periodic introduction of CWD-naïve animals to Site 2 was not part of this study, and animals were utilized for a variety of research purposes over many decades. These animals were not always introduced into pre-existing CWD-positive herds but were often kept isolated in enclosures that had previously housed CWD-positive animals; however, contact between groups through gates and pens routinely occurred unless specific isolation protocols were in place for research projects. Animals with the codon 132 MM genotype typically reside on site for 2 to 7 years before becoming clinical for CWD. Animals with the ML genotype tended to survive longer and had an average lifespan of four to 9 years [26,27].

##### CWD isolate study animals

The brains of four CWD-infected terminal stage adult females of unknown age [2-A, 2-B, 2-C, 2-D] from this site were subjected to biochemical analysis (Table 2).

##### Introduction of naïve animals

Elks were added to the site over a 60-year time period for various reasons and from multiple sources, including wild-captured adults from within the same state, such as orphaned neonates, 6–8 month old elk calves, or natural additions from the resident population. Naïve animals introduced to this site were not part of the study and are mentioned simply to increase understanding of the location history.

### Sequencing of functional PrP genes

Genomic DNA was purified from frozen brain tissues using a standard phenol-chloroform extraction procedure. The purified DNA samples were subjected to two more rounds of phenol/chloroform extraction to remove any residual prions before further manipulations. The PrP-coding region from functional PrP genes was amplified by PCR (95°C for 4 min; 35 cycles of 95°C for 30 sec, 54°C for 30 sec and 72°C for 60 sec; followed by 72°C for 10 min) using the Phusion high fidelity polymerase (New England Biolabs, Ipswich, MA, USA) and primers DePrP223 (ACACCCTCTTTATTTTGCAG) and DePrP224 (AGAAGATAATGAAAACAGGAAG) that had been previously phosphorylated by T4 polynucleotide kinase (New England Biolabs, Ipswich, MA, USA). The PCR products were recovered after gel electrophoresis in 1% agarose gels using the QIAquick gel extraction kit (Qiagen, Hilden, Germany), cloned into the EcoRV site of pstBlue-1, transformed into *E coli*. and the plasmid DNA extracted with the PureLink Quick Plasmoid Miniprep Kit (Invitrogen. Carlsbad, CA, USA). The purified PCR products and the cloned plasmids were subjected to sequencing by MCLAB (South San Francisco, CA, USA) using primers DePRP223 and DePrP224. The final PrP genotypes were determined based on a combination of sequencing traces from the PCR products and sequences of multiple clones from each elk DNA sample.

### Fibril stability assay

The IDEXX HerdChek BSE-Scrapie Antigen EIA test kit selectively binds and detects PrP^RES^ as opposed to the normal endogenous cellular prion protein (PrP^C^) or unfolded PrP. We used this kit to measure the amount of PrP^RES^ in tissue samples after incubation in GdnHCl as described in Vrentas *et al*. [9]. Briefly, the brain stem samples stored at −80°C were homogenized at 20% (w/v) in 1 x PBS (Dulbecco , pH 7.4, no calcium and magnesium) and incubated in 0.25–4.0 M GdnHCl, 1 x PBS for 1 hr. The amount of brain homogenate used in each sample was adjusted to ensure that the final optical density at 450 nm (OD450) of the EIA was at or near 1.0 and involved a minimum of 1 to 10 dilutions into the buffered GdnHCl containing solution. Following the 1-hr incubation, all samples were diluted with PBS for a final GdnHCl concentration of 0.25 M and loaded onto the EIA test kit plates. Sample analysis subsequently followed the manufacturer’s ‘short protocol’ for small ruminants. OD450 values for each sample were determined and normalized to either the 0.25 M or 1.0 M sample following subtraction of the IDEXX kit provided negative control. The unfolding curve for each animal was repeated 3 times, the C_mid_ (denaturant concentration at the midpoint of unfolding) determined by visual inspection, and the mean for each determined. The average C_mid_ for each site is then determined as the average across animals. A two-tailed t-test was used to determine the significance between the unfolding curves from each site.

### Western blot analysis

The brain samples were homogenized at 20% in 1x PBS (pH 7.4, without calcium and magnesium) and stored at −80°C before examination. Before the Western blot, the frozen 20% brain homogenate was thawed; 50ul was taken, mixed with an equal volume of 2x lysis buffer [200 mM NaCl, 200 mM Tris-HCL (pH 8.0), 20 mM EDTA, 1% NP-40, 1% sodium deoxycholate] to get 10% brain homogenate in 1x lysis buffer, which was then incubated at −80°C for at least 12 hours. After thawing on ice, the 10% brain homogenate in the lysis buffer was briefly sonicated (about 10 s), centrifuged for 8 minutes at 500 g at 4°C, and the supernatant carefully transferred to a fresh tube. Aliquot of the supernatant (1–2.5 ul for elk samples and 15ul for human sCJD type 1 and type 2 controls) was taken, and 10% brain homogenate from FVB/PrPKO mice was added to the elk sample tubes to obtain a total of 15 ul of brain homogenate for each sample tube; this was done to ensure highly similar total protein contents in each sample tube for the subsequent proteinase K (PK) digestion and comparable PrP^Sc^ signals for all samples. PK was added to a final concentration of 100 µg/ml, and the digestion was performed for 1 hr at 37°C in a heat-block, with vigorous shaking. In the addition of 15 ul of Tricine loading buffer (BioRad, Cat. No. 1,610,739), the samples were boiled at 100°C for 10 min, separated on 16.5% Criterion Tris-Tricine SDS-PAGE gels (Biorad, Cat. No. 3,450,064) and transferred onto a polyvinylidene difluoride (PVDF) membrane (Millipore, Billerica, MA) for 2 h at 0.5 A. The membranes were probed with anti-PrP primary antibodies (8H4, 1:5000; 9A2, 1:4000; or POM19, 1:500) and sheep anti-mouse IgG conjugated with horseradish peroxidase (Amersham, Cat. No. NA931) as the secondary antibody and developed with the ECL2 Western blotting substrate (Thermo Scientific, PI80196) for 5 minutes and then developed using Carestream Biomax Xar film (Sigma 165–1454).

## Results

### Sequencing of functional PrP genes in the elk samples

The functional PrP gene sequences in the elk samples were determined by sequencing the PCR product of the PrP ORFs and multiple clones of the PrP ORF PCR products for each sample. Site 1 animal A was methionine/leucine (ML), and animal B was methionine/methionine (MM) at codon 132. Site 2 animals A and B were MM, while C and D were ML at codon 132 (Table 2). Introduced naïve animals to Site 2 were not part of this study, and their genetics are unknown.

### Direct and indirect contact groups on site 1

A year after its introduction, the female, MM, direct contact elk (S13) was found to be CWD-positive by rectal biopsy and was euthanized. The ‘native’ 11-year-old female that she was co-housed with was also found to be CWD-positive by rectal biopsy at the same time. Neither was clinical. Three years after the introduction, an asymptomatic, direct contact, MM male (801 T) was found to be CWD positive by rectal biopsy. After 5 years, the introduction aspect of the study at Site 1 was terminated. All-remaining animals, who were in excellent body condition and showed no clinical signs of CWD, were euthanized, necropsied, and tested for CWD by IHC. All the remaining males (indirect group n = 6 and direct group n = 6) were non-detect for CWD by IHC in medial retropharyngeal lymph nodes and obex, the only tissues examined.

### Fibril stability

**Figure 1.**
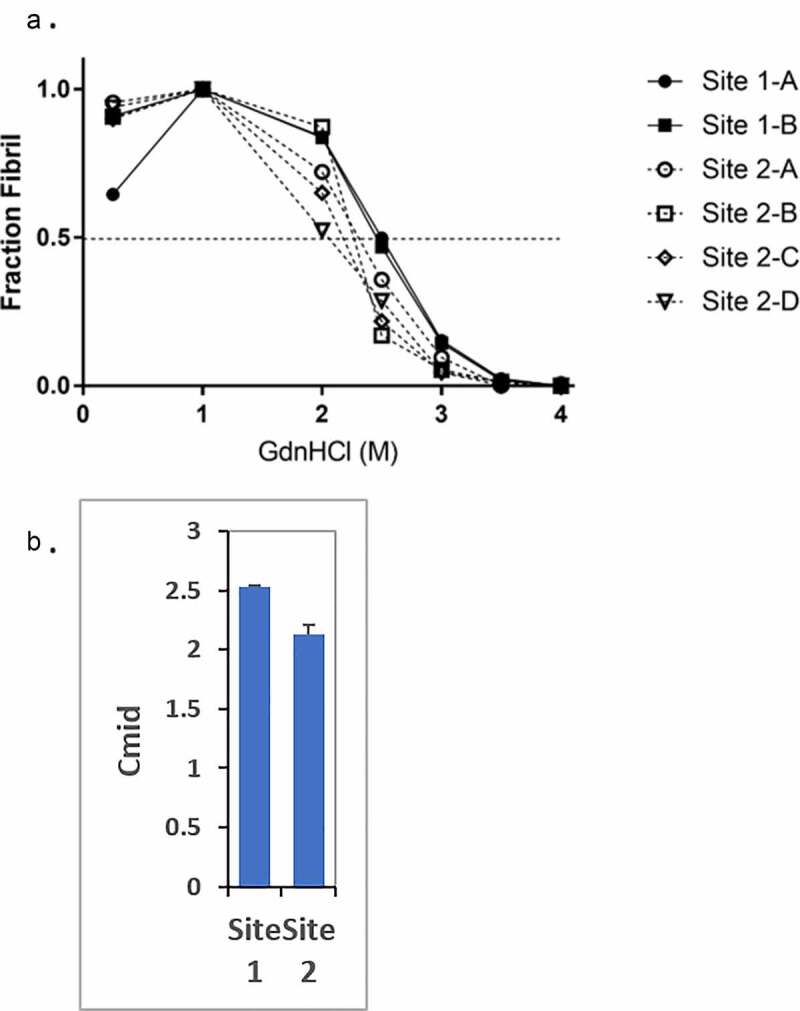
The fibril stability of PrP^Sc^ from the two sites are different. (a) Representative fibril stability curves for samples from sites 1 and 2. The connecting lines are presented to aid the eye and highlight the point at which each unfolding curve intersects a fraction fibril of 0.5. Both Site 1-A and Site 1-B samples exhibit visibly higher fibril stability than the Site 2 samples based upon the concentration of GdnHCl at which the fraction fibril reaches 0.5 (Cmid). (b) Bargraph of quantified Cmid values of the site 1 and site 2 samples. The average Cmid value of site 1 samples (2.53 ± 0.05 M) is significantly higher than that of site 2 samples (2.16 ± 0.09 M) (p = 0.0043)

The fibril stability assay indicated that Site 1 animal samples had a discernibly higher resistance to unfolding by the chemical denaturant GdnHCl when compared with those of Site 2 animal samples, with the mean midpoint of the unfolding curves at 2.53 M and 2.12 M, respectively (Figure 1). The difference in fibril stability between prions in animals from the two sites is statistically significant (p = 0.0043) (Figure 1b).

### Western blot

**Figure 2.**
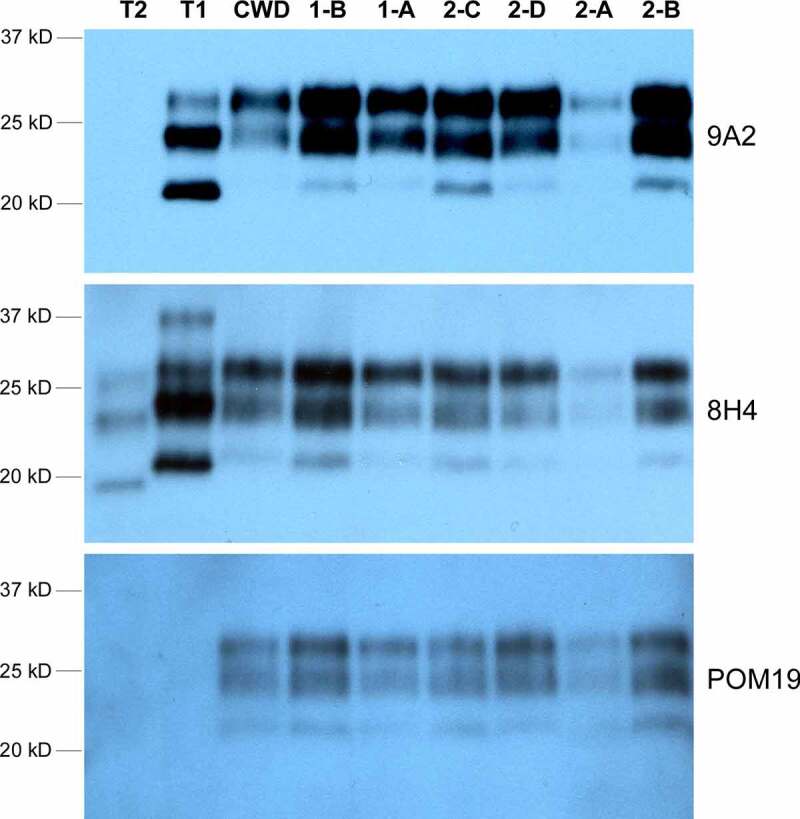
Western blot of CWD PrP^RES^ in the brain tissues from Sites 1 and 2 elk. Four anti-PrP antibodies that target different PrP epitopes were used: 9A2 (epitope 97–115), 8H4 (epitope 177–180), and POM19 (epitope 201–225). POM19 does not recognize human PrP whereas 9A2 shows higher affinity for type 1 PrP^RES^. T1 and T2 are type 1 sCJD and type 2 sCJD human brain tissue controls, respectively. ‘CWD’ is a positive control elk CWD brain sample from an unrelated study. The loading amount was adjusted between the brain samples to allow more comparable signal strength for all samples with the same exposure: 5ul of 5% brain homogenate for 1-B, 1-A, 2-C and 2-A; 2ul of 5% brain homogenate for 2-D, 2-B and the control ‘CWD’ samples; 30ul of 5% brain homogenate for T1 and T2 human sCJD controls. The difference in sample loading volume was made up with 5% brain homogenate from PrPKO/FVB mice

The band pattern of the CWD PrP^RES^ in Western Blot was indistinguishable between the two groups when probed with three different antibodies that target different regions of the PrP protein (Figure 2).

## Discussion

The observation that CWD appears to behave differently between two naturally infected captive elk populations precipitated a biochemical analysis to look for biochemical differences in the CWD prion proteins present in animals on these sites.

One biochemical method of differentiating prion isolates or strains is to evaluate the stability of the aberrant prion protein’s structural conformation utilizing a denaturing agent such as GdnHCl. The more stable a conformation, the greater the concentration of GdnHCl required to denature the protein. Previous work has shown that TSEs with greater prion stability exhibited longer incubation periods than those with lower stability when bioassayed experimentally in the laboratory [9,28,29]. Conversely, a study utilizing hamsters reported the opposite, with the more stable PrP^RES^ conveying a shorter incubation period [7]. Site 1 had a history of a low prevalence rate of CWD with one or two animals diagnosed per year (5–10%), and a CWD isoform with greater conformational stability as compared to Site 2. The relatively low prevalence rate does not appear to be a result of an increased incubation period, but rather a limited attack rate as illustrated by the direct and indirect contact findings. Six of the seven naïve elk co-housed with the resident herd remained free of CWD 5 years after its introduction. In contrast, Site 2 had a higher prevalence of CWD cases with all elk eventually becoming CWD positive with a CWD isoform that was less stable. The difference in history and timeline of CWD between the two facilities must be noted; however, as there was longer-term CWD contamination and accumulation at Site 2. While high animal and environmental CWD burden may be responsible for this level of CWD prevalence, it does not account for the difference in prion conformational stability. It has been hypothesized that prion strains may change over time. At site 2, perhaps a more infectious isoform(s)/strain(s) may have emerged at some point and become dominant at the site over time. The data from this study seem to support the above notion considering CWD positive animals had been housed at Site 2 for decades and had a high level of transmission. More focused studies will need to be done to explore this idea further.

Animal 2-B had a different denaturation pattern than the other Site 2 CWD samples. Animal 2-B’s CWD remained stable until the GdnHCl concentration reached 2 M, after which there was a precipitous decline in stability. In contrast, the other three Site 2 CWD samples began to lose stability at 1 M and continued to lose stability in a less abrupt manner. The significance of this difference is unclear.

Western blot analysis of the samples did not reveal any significant molecular weight or band pattern changes between the CWD isoforms despite utilizing three different anti-PrP antibodies. These findings demonstrate that different prion isolates can behave very similarly in Western Blots.

Genetic polymorphisms in codon 132 of the prion protein gene (prnp) have been shown to strongly influence the proliferation and incubation period of CWD in elk [30,31]. The most common genotype in wild elk at codon 132 is methionine/methionine (MM) [32]. These animals have decreased incubation times and thus faster disease proliferation than methionine/leucine (ML) or leucine/leucine (LL) genotypes. However, genotypes at codon 132, MM or ML, appeared to have no influence on the stability of prion fibrils in this study, with stability differing by site rather than by genotype. Of the surviving 12 animals in the direct and indirect contact study at Site 1, 10 were MM at codon 132. In contrast, CWD positive MM animals from Site 2 had a typical life span of two to 3 years. In a study examining different CWD strains in Syrian golden hamsters, the authors concluded that the observed differences in the prion strain in their model were independent of prion gene polymorphisms [28]. Our findings are consistent with this and those of Moore, *et al*. who found no difference in fibril stability between experimentally CWD-infected MM and ML elk [31].

In this study, we use the term isolate to describe our findings versus strain, as we were unable to conduct transgenic mouse bioassay, and lacked tissues to compare brain and organ pathology to address all the previously described criteria for a prion strain, such as variations in clinical signs, incubation period, distribution of the misfolded prion (PrP^RES^) in the body, and the stability of the PrP^RES^ protein within the same species and TSE type. Despite this, we feel that the data we have strongly suggest the presence of two different CWD strains.

It is clear from looking at the literature that the phenomenon of strains or isolates in prion diseases is complex. Studies have shown that multiple prion strains can be present in a single sample and that the ratio of strains presents in the sample may alter which form becomes dominant [10,12,33,34]. Furthermore, multiple strains can sometimes interfere with one another, resulting in the dominance of one type over another [35–37], and interaction between infectious prions and soil can alter which form becomes dominant over time, suggesting a role for environmental factors in this selection process [38]. Both the *cloud hypothesis* and the *deformed template theory* of prion propagation suggest that the replication environment ultimately determines the final confirmation of *the de novo* prions by either making conditions more conducive to one strain over the other (*cloud hypothesis*) or by directly influencing the *de novo* development itself (*deformed template theory*) [39,40]. The replication environment referred to in these hypotheses is the region of replication within the body of the animal. Previous work conducted in our laboratory, utilizing a cervidized transgenic mouse model, demonstrated that altering this internal environment, by increasing or decreasing copper consumption, affected neuroinflammatory gene expression and the CWD incubation period in the mice [41]. It is unclear if exogenous factors, such as cation ingestion or soil type, played a role in the development of the different isoforms we detected.

Because the sample numbers in this study were low, stronger conclusions could not be drawn. However, our findings suggest that prion isoforms may influence the infectivity of the CWD prion and thus how the disease behaves in the landscape. It is our hope that these findings add another piece to the puzzle of CWD prion diversity and the potential evolution of CWD prions over time. More research is needed to understand the role and significance of CWD isoforms in disease transmission.
